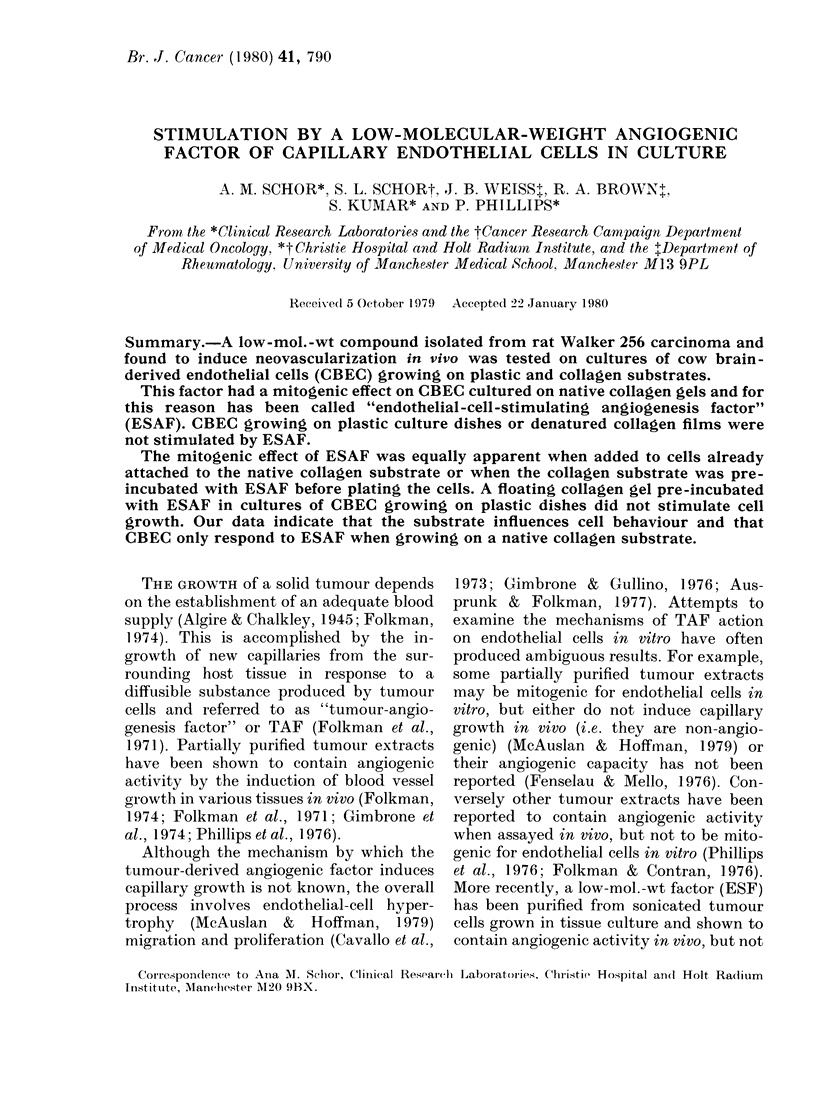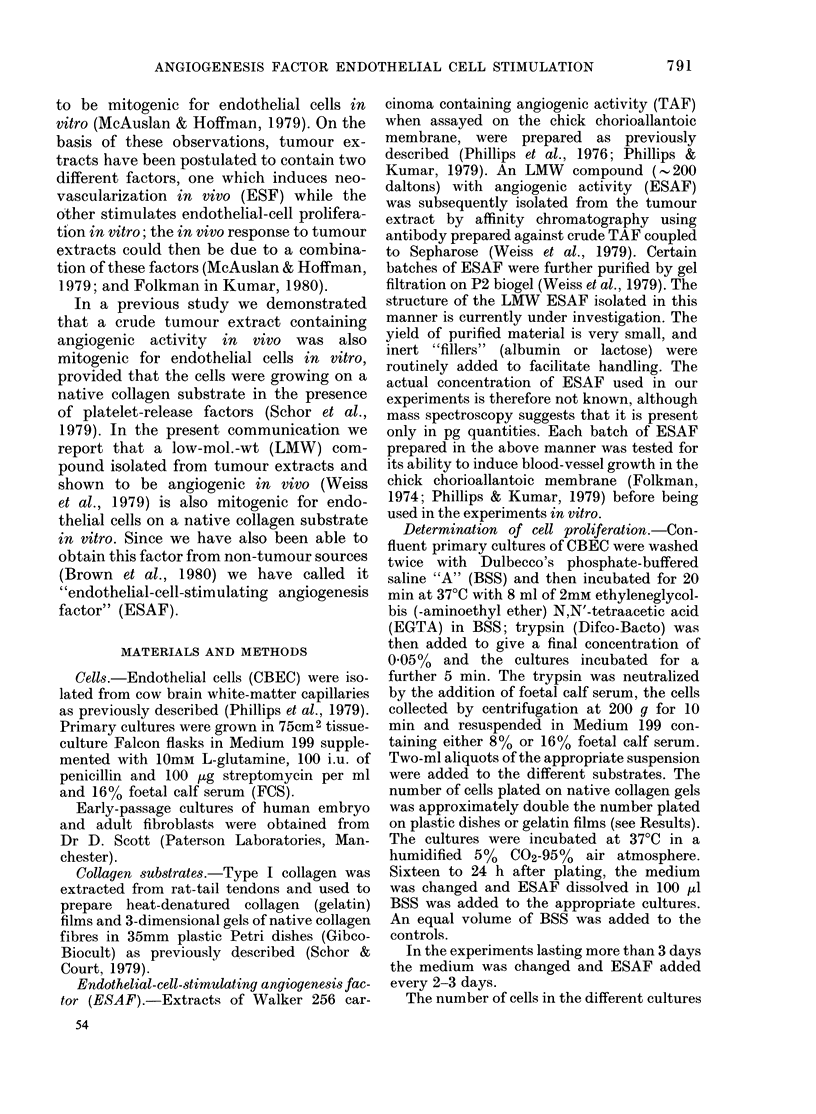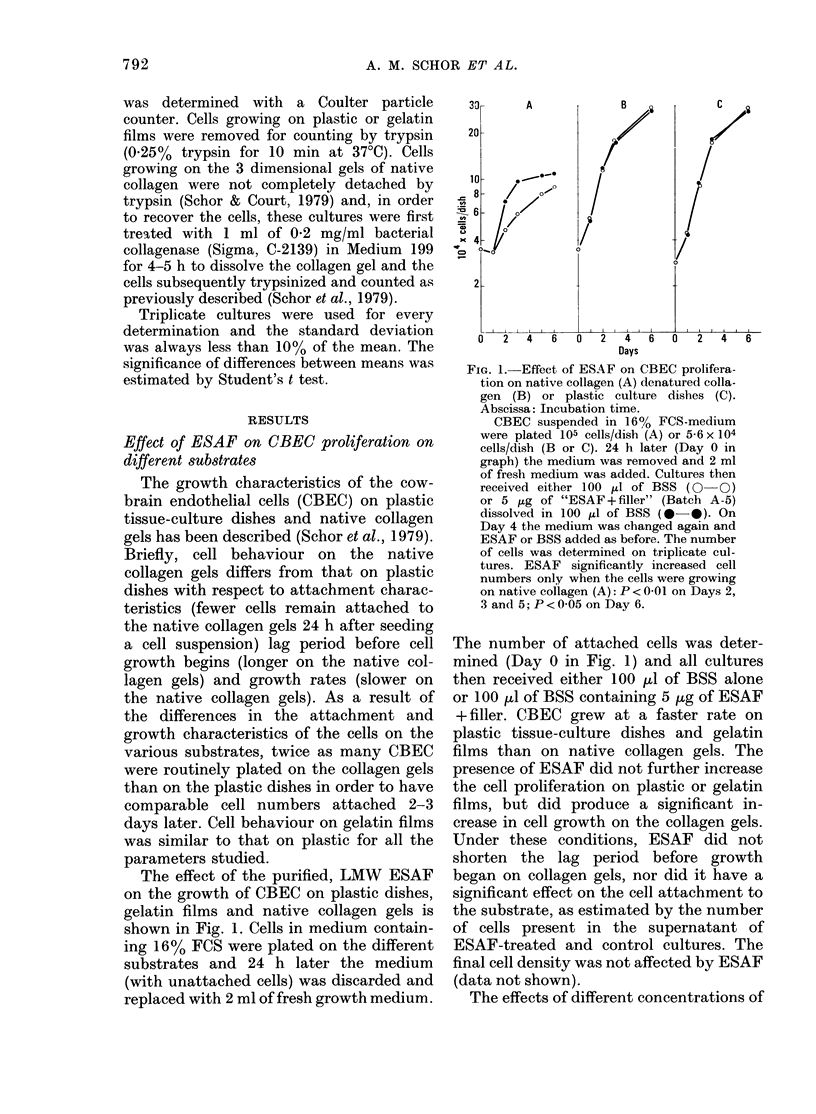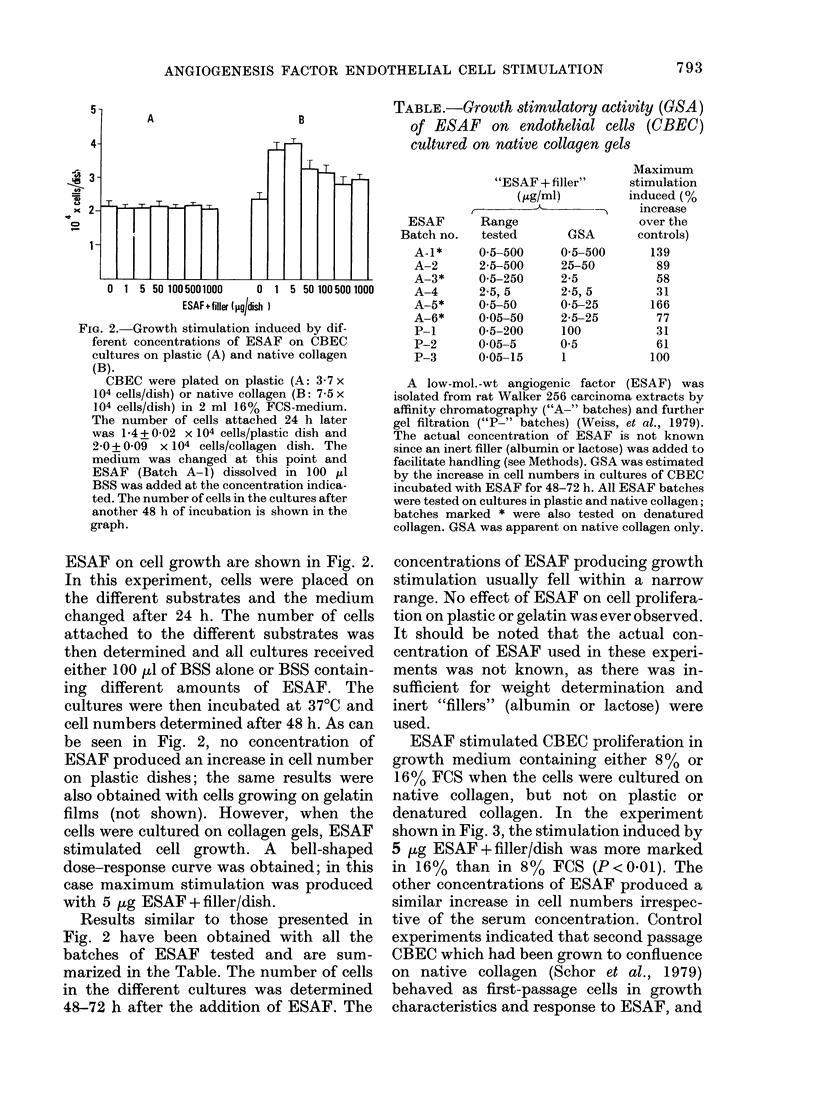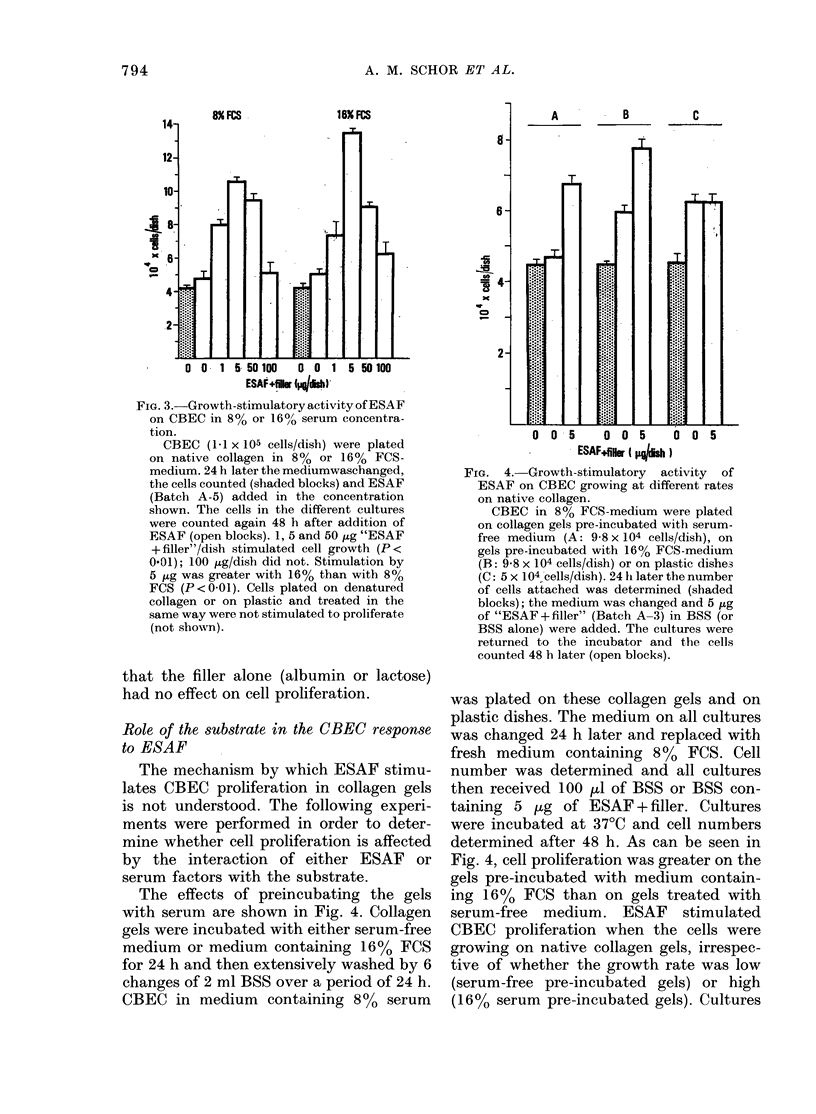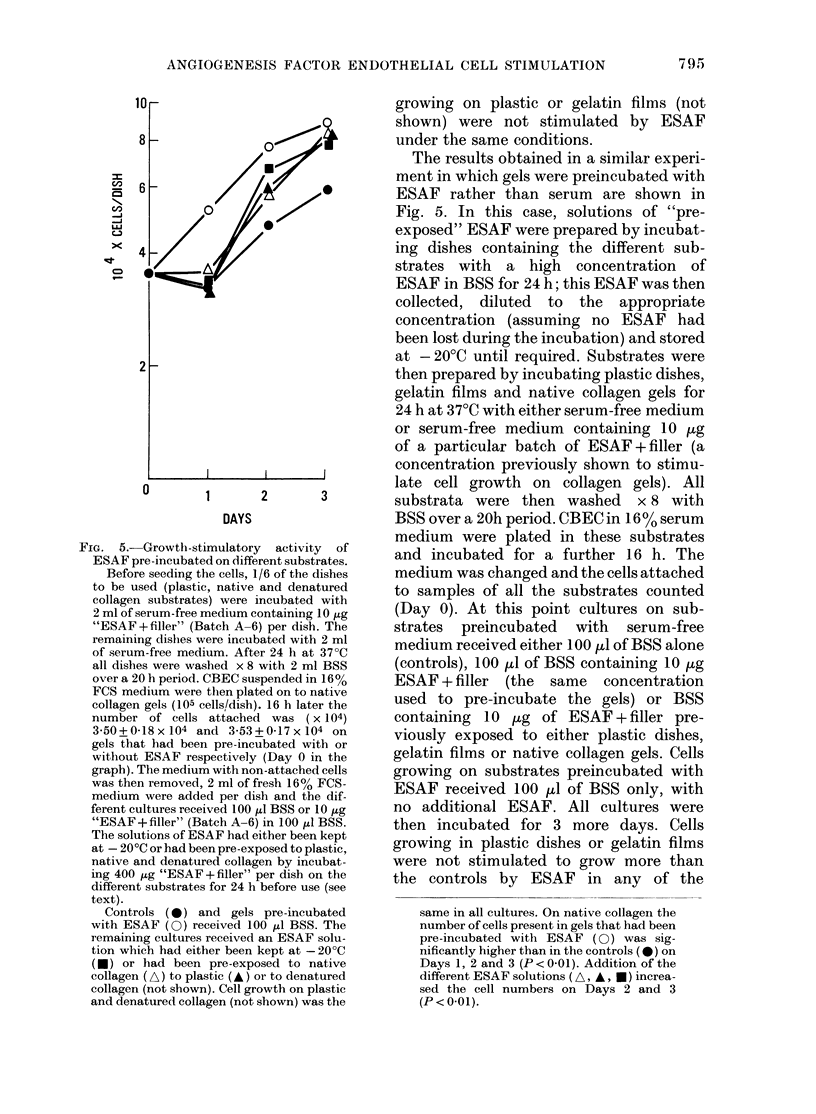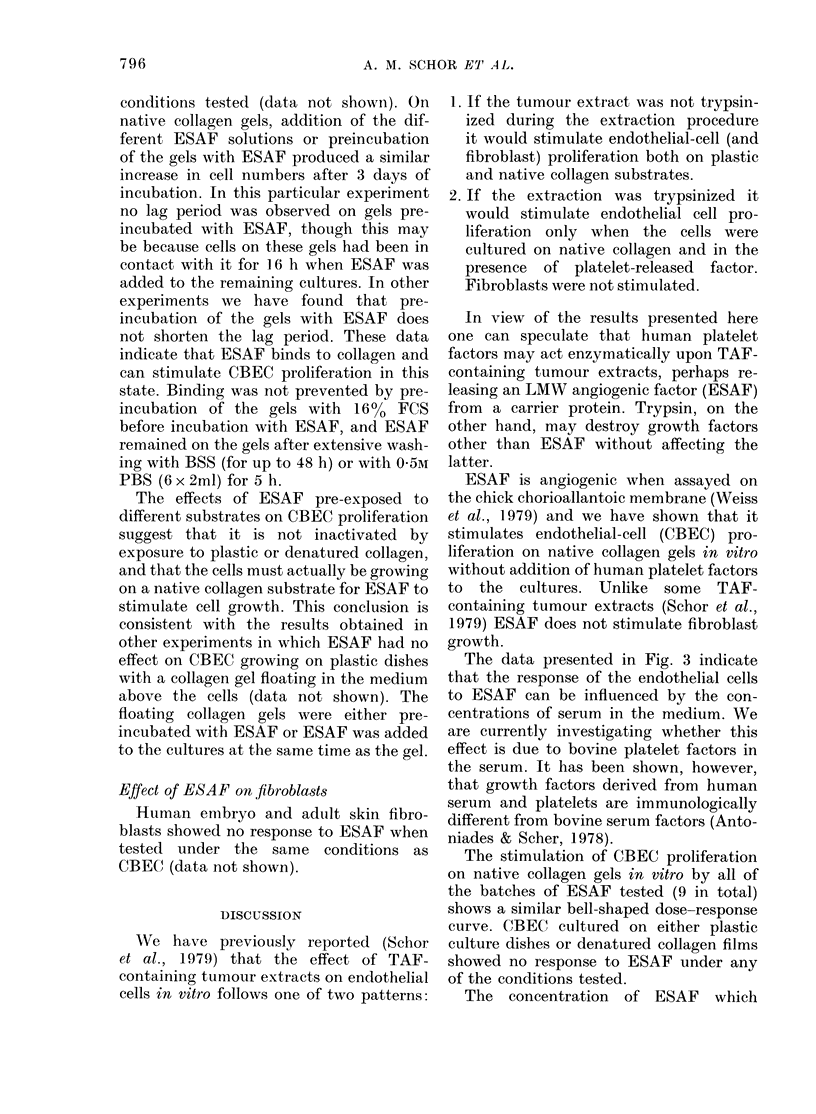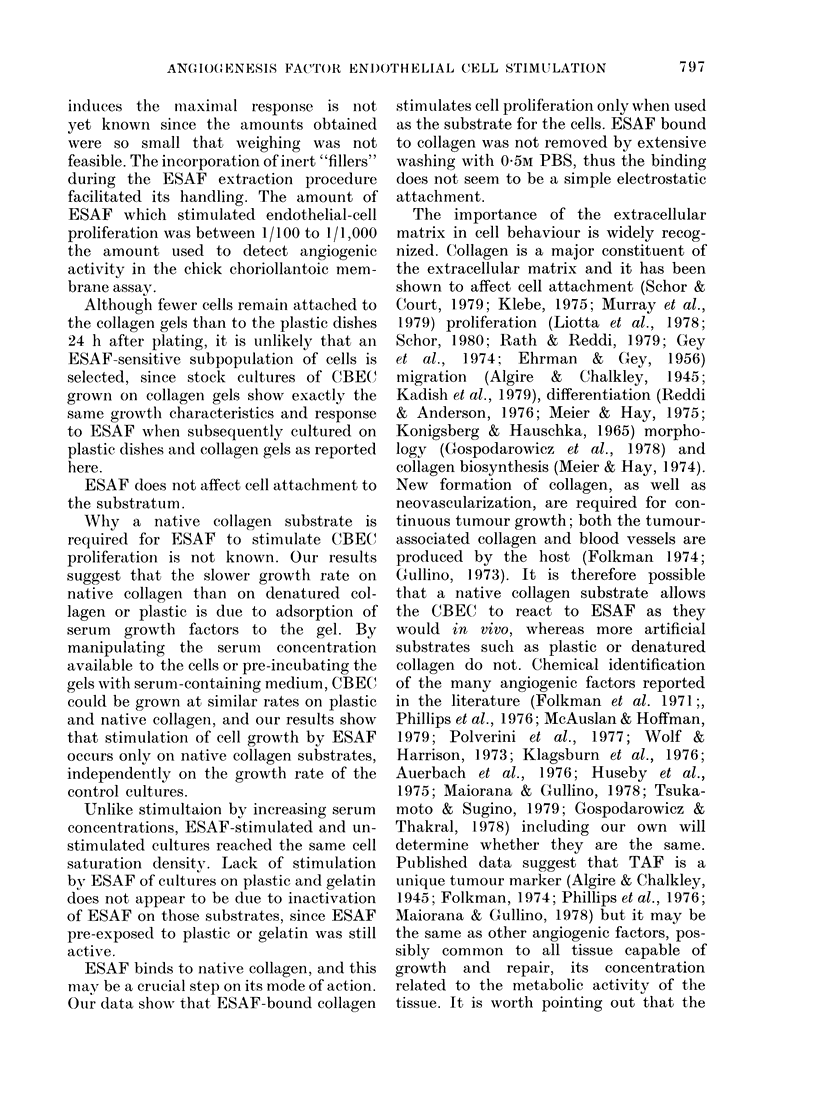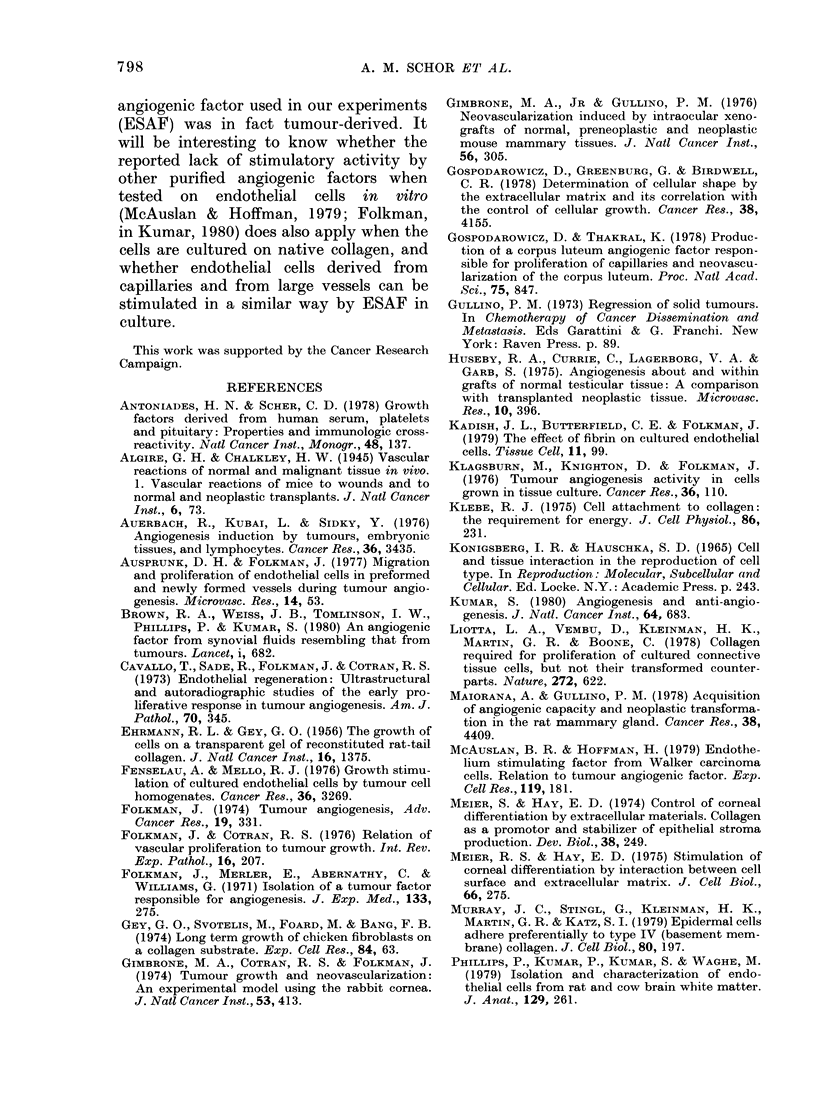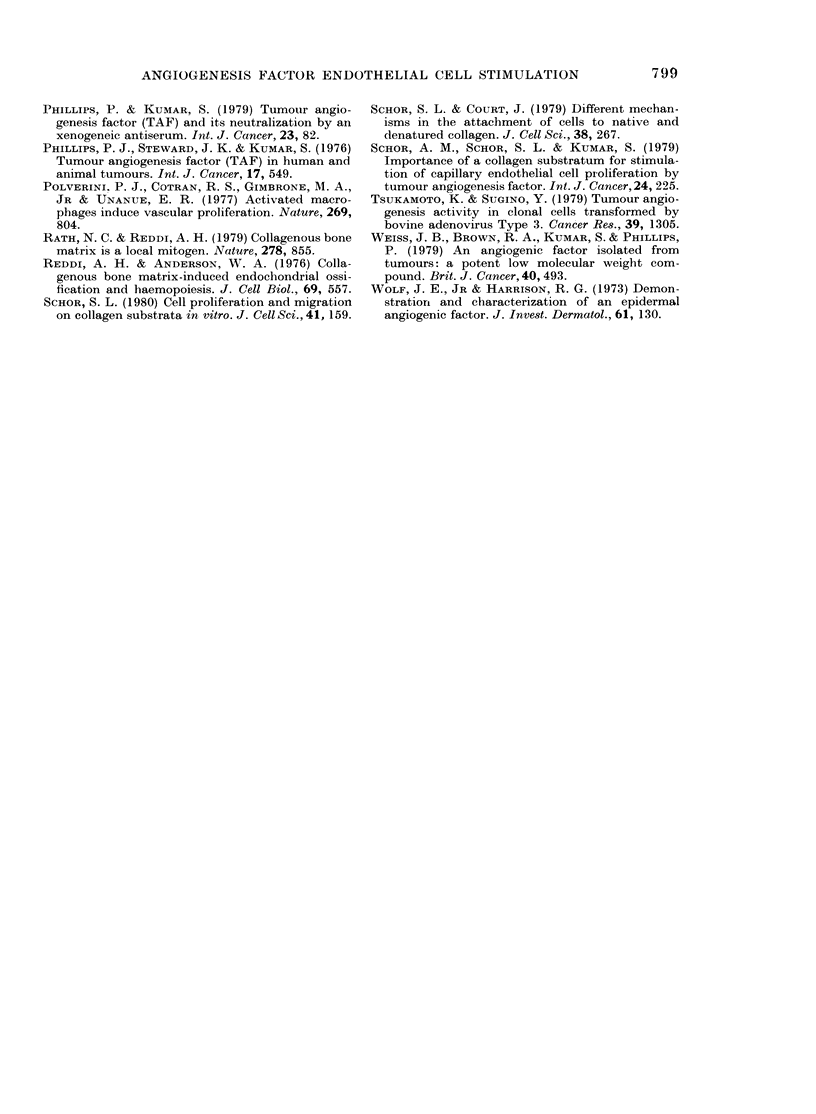# Stimulation by a low-molecular-weight angiogenic factor of capillary endothelial cells in culture.

**DOI:** 10.1038/bjc.1980.143

**Published:** 1980-05

**Authors:** A. M. Schor, S. L. Schor, J. B. Weiss, R. A. Brown, S. Kumar, P. Phillips

## Abstract

A low-mol.-wt compound isolated from rat Walker 256 carcinoma and found to induce neovascularization in vivo was tested on cultures of cow brain-derived endothelial cells (CBEC) growing on plastic and collagen substrates. This factor had a mitogenic effect on CBEC cultured on native collagen gels and for this reason has been called "endothelial-cell-stimulating angiogenesis factor" (ESAF). CBEC growing on plastic culture dishes or denatured collagen films were not stimulated by ESAF. The mitogenic effect of ESAF was equally apparent when added to cells already attached to the native collagen substrate or when the collagen substrate was pre-incubated with ESAF before plating the cells. A floating collagen gel pre-incubated with ESAF in cultures of CBEC growing on plastic dishes did not stimulate cell growth. Our data indicate that the substrate influences cell behaviour and that CBEC only respond to ESAF when growing on a native collagen substrate.


*Br. J. Cancer* (1980) **41**, 790

### STIMULATION BY A LOW-MOLECULAR-WEIGHT ANGIOGENIC FACTOR OF CAPILLARY ENDOTHELIAL CELLS IN CULTURE

A. M. SCHOR*, S. L. SCHOR†, J. B. WEISS‡, R. A. BROWN‡, S. KUMAR* AND P. PHILLIPS*

*From the *Clinical Research Laboratories and the †Cancer Research Campaign Department of Medical Oncology, *†Christie Hospital and Holt Radium Institute, and the ‡Department of Rheumatology, University of Manchester Medical School, Manchester M13 9PL*

Received 5 October 1979 Accepted 22 January 1980

**Summary.—A low-mol.-wt compound isolated from rat Walker 256 carcinoma and found to induce neovascularization *in vivo* was tested on cultures of cow brain-derived endothelial cells (CBEC) growing on plastic and collagen substrates.**

**This factor had a mitogenic effect on CBEC cultured on native collagen gels and for this reason has been called "endothelial-cell-stimulating angiogenesis factor" (ESAF). CBEC growing on plastic culture dishes or denatured collagen films were not stimulated by ESAF.**

**The mitogenic effect of ESAF was equally apparent when added to cells already attached to the native collagen substrate or when the collagen substrate was pre-incubated with ESAF before plating the cells. A floating collagen gel pre-incubated with ESAF in cultures of CBEC growing on plastic dishes did not stimulate cell growth. Our data indicate that the substrate influences cell behaviour and that CBEC only respond to ESAF when growing on a native collagen substrate.**

THE GROWTH of a solid tumour depends on the establishment of an adequate blood supply (Algire & Chalkley, 1945; Folkman, 1974). This is accomplished by the in-growth of new capillaries from the surrounding host tissue in response to a diffusible substance produced by tumour cells and referred to as "tumour-angiogenesis factor" or TAF (Folkman *et al.*, 1971). Partially purified tumour extracts have been shown to contain angiogenic activity by the induction of blood vessel growth in various tissues *in vivo* (Folkman, 1974; Folkman *et al.*, 1971; Gimbrone *et al.*, 1974; Phillips *et al.*, 1976).

Although the mechanism by which the tumour-derived angiogenic factor induces capillary growth is not known, the overall process involves endothelial-cell hypertrophy (McAuslan & Hoffman, 1979) migration and proliferation (Cavallo *et al.*, 1973; Gimbrone & Gullino, 1976; Ausprunk & Folkman, 1977). Attempts to examine the mechanisms of TAF action on endothelial cells *in vitro* have often produced ambiguous results. For example, some partially purified tumour extracts may be mitogenic for endothelial cells *in vitro*, but either do not induce capillary growth *in vivo* (*i.e.* they are non-angiogenic) (McAuslan & Hoffman, 1979) or their angiogenic capacity has not been reported (Fenselau & Mello, 1976). Conversely other tumour extracts have been reported to contain angiogenic activity when assayed *in vivo*, but not to be mitogenic for endothelial cells *in vitro* (Phillips *et al.*, 1976; Folkman & Contran, 1976). More recently, a low-mol.-wt factor (ESF) has been purified from sonicated tumour cells grown in tissue culture and shown to contain angiogenic activity *in vivo*, but not

Correspondence to Ana M. Schor, Clinical Research Laboratories, Christie Hospital and Holt Radium Institute, Manchester M20 9BX.

to be mitogenic for endothelial cells *in vitro* (McAuslan & Hoffman, 1979). On the basis of these observations, tumour extracts have been postulated to contain two different factors, one which induces neovascularization *in vivo* (ESF) while the other stimulates endothelial-cell proliferation *in vitro*; the *in vivo* response to tumour extracts could then be due to a combination of these factors (McAuslan & Hoffman, 1979; and Folkman in Kumar, 1980).

In a previous study we demonstrated that a crude tumour extract containing angiogenic activity *in vivo* was also mitogenic for endothelial cells *in vitro*, provided that the cells were growing on a native collagen substrate in the presence of platelet-release factors (Schor *et al.*, 1979). In the present communication we report that a low-mol.-wt (LMW) compound isolated from tumour extracts and shown to be angiogenic *in vivo* (Weiss *et al.*, 1979) is also mitogenic for endothelial cells on a native collagen substrate *in vitro*. Since we have also been able to obtain this factor from non-tumour sources (Brown *et al.*, 1980) we have called it "endothelial-cell-stimulating angiogenesis factor" (ESAF).

### MATERIALS AND METHODS

*Cells.*—Endothelial cells (CBEC) were isolated from cow brain white-matter capillaries as previously described (Phillips *et al.*, 1979). Primary cultures were grown in 75cm$^2$ tissue-culture Falcon flasks in Medium 199 supplemented with 10mM L-glutamine, 100 i.u. of penicillin and 100 $\mu$g streptomycin per ml and 16% foetal calf serum (FCS).

Early-passage cultures of human embryo and adult fibroblasts were obtained from Dr D. Scott (Paterson Laboratories, Manchester).

*Collagen substrates.*—Type I collagen was extracted from rat-tail tendons and used to prepare heat-denatured collagen (gelatin) films and 3-dimensional gels of native collagen fibres in 35mm plastic Petri dishes (Gibco-Biocult) as previously described (Schor & Court, 1979).

*Endothelial-cell-stimulating angiogenesis factor (ESAF).*—Extracts of Walker 256 carcinoma containing angiogenic activity (TAF) when assayed on the chick chorioallantoic membrane, were prepared as previously described (Phillips *et al.*, 1976; Phillips & Kumar, 1979). An LMW compound ($\sim$200 daltons) with angiogenic activity (ESAF) was subsequently isolated from the tumour extract by affinity chromatography using antibody prepared against crude TAF coupled to Sepharose (Weiss *et al.*, 1979). Certain batches of ESAF were further purified by gel filtration on P2 biogel (Weiss *et al.*, 1979). The structure of the LMW ESAF isolated in this manner is currently under investigation. The yield of purified material is very small, and inert "fillers" (albumin or lactose) were routinely added to facilitate handling. The actual concentration of ESAF used in our experiments is therefore not known, although mass spectroscopy suggests that it is present only in pg quantities. Each batch of ESAF prepared in the above manner was tested for its ability to induce blood-vessel growth in the chick chorioallantoic membrane (Folkman, 1974; Phillips & Kumar, 1979) before being used in the experiments *in vitro*.

*Determination of cell proliferation.*—Confluent primary cultures of CBEC were washed twice with Dulbecco's phosphate-buffered saline "A" (BSS) and then incubated for 20 min at 37°C with 8 ml of 2mM ethyleneglycol-bis (-aminoethyl ether) N,N′-tetraacetic acid (EGTA) in BSS; trypsin (Difco-Bacto) was then added to give a final concentration of 0·05% and the cultures incubated for a further 5 min. The trypsin was neutralized by the addition of foetal calf serum, the cells collected by centrifugation at 200 *g* for 10 min and resuspended in Medium 199 containing either 8% or 16% foetal calf serum. Two-ml aliquots of the appropriate suspension were added to the different substrates. The number of cells plated on native collagen gels was approximately double the number plated on plastic dishes or gelatin films (see Results). The cultures were incubated at 37°C in a humidified 5% $CO_2$-95% air atmosphere. Sixteen to 24 h after plating, the medium was changed and ESAF dissolved in 100 $\mu$l BSS was added to the appropriate cultures. An equal volume of BSS was added to the controls.

In the experiments lasting more than 3 days the medium was changed and ESAF added every 2–3 days.

The number of cells in the different cultures was determined with a Coulter particle counter. Cells growing on plastic or gelatin films were removed for counting by trypsin (0·25% trypsin for 10 min at 37°C). Cells growing on the 3 dimensional gels of native collagen were not completely detached by trypsin (Schor & Court, 1979) and, in order to recover the cells, these cultures were first treated with 1 ml of 0·2 mg/ml bacterial collagenase (Sigma, C-2139) in Medium 199 for 4–5 h to dissolve the collagen gel and the cells subsequently trypsinized and counted as previously described (Schor *et al.*, 1979).

Triplicate cultures were used for every determination and the standard deviation was always less than 10% of the mean. The significance of differences between means was estimated by Student's *t* test.

FIG. 1.—Effect of ESAF on CBEC proliferation on native collagen (A) denatured collagen (B) or plastic culture dishes (C). Abscissa: Incubation time.

CBEC suspended in 16% FCS-medium were plated $10^5$ cells/dish (A) or $5{\cdot}6 \times 10^4$ cells/dish (B or C). 24 h later (Day 0 in graph) the medium was removed and 2 ml of fresh medium was added. Cultures then received either 100 $\mu$l of BSS (○—○) or 5 $\mu$g of "ESAF+filler" (Batch A-5) dissolved in 100 $\mu$l of BSS (●—●). On Day 4 the medium was changed again and ESAF or BSS added as before. The number of cells was determined on triplicate cultures. ESAF significantly increased cell numbers only when the cells were growing on native collagen (A): $P < 0{\cdot}01$ on Days 2, 3 and 5; $P < 0{\cdot}05$ on Day 6.

### RESULTS

### *Effect of ESAF on CBEC proliferation on different substrates*

The growth characteristics of the cow-brain endothelial cells (CBEC) on plastic tissue-culture dishes and native collagen gels has been described (Schor *et al.*, 1979). Briefly, cell behaviour on the native collagen gels differs from that on plastic dishes with respect to attachment characteristics (fewer cells remain attached to the native collagen gels 24 h after seeding a cell suspension) lag period before cell growth begins (longer on the native collagen gels) and growth rates (slower on the native collagen gels). As a result of the differences in the attachment and growth characteristics of the cells on the various substrates, twice as many CBEC were routinely plated on the collagen gels than on the plastic dishes in order to have comparable cell numbers attached 2–3 days later. Cell behaviour on gelatin films was similar to that on plastic for all the parameters studied.

The effect of the purified, LMW ESAF on the growth of CBEC on plastic dishes, gelatin films and native collagen gels is shown in Fig. 1. Cells in medium containing 16% FCS were plated on the different substrates and 24 h later the medium (with unattached cells) was discarded and replaced with 2 ml of fresh growth medium. The number of attached cells was determined (Day 0 in Fig. 1) and all cultures then received either 100 $\mu$l of BSS alone or 100 $\mu$l of BSS containing 5 $\mu$g of ESAF +filler. CBEC grew at a faster rate on plastic tissue-culture dishes and gelatin films than on native collagen gels. The presence of ESAF did not further increase the cell proliferation on plastic or gelatin films, but did produce a significant increase in cell growth on the collagen gels. Under these conditions, ESAF did not shorten the lag period before growth began on collagen gels, nor did it have a significant effect on the cell attachment to the substrate, as estimated by the number of cells present in the supernatant of ESAF-treated and control cultures. The final cell density was not affected by ESAF (data not shown).

The effects of different concentrations of

FIG. 2.—Growth stimulation induced by different concentrations of ESAF on CBEC cultures on plastic (A) and native collagen (B).

CBEC were plated on plastic (A: $3 \cdot 7 \times 10^4$ cells/dish) or native collagen (B: $7 \cdot 5 \times 10^4$ cells/dish) in 2 ml 16% FCS-medium. The number of cells attached 24 h later was $1 \cdot 4 \pm 0 \cdot 02 \times 10^4$ cells/plastic dish and $2 \cdot 0 \pm 0 \cdot 09 \times 10^4$ cells/collagen dish. The medium was changed at this point and ESAF (Batch A-1) dissolved in 100 μl BSS was added at the concentration indicated. The number of cells in the cultures after another 48 h of incubation is shown in the graph.

TABLE.—*Growth stimulatory activity (GSA) of ESAF on endothelial cells (CBEC) cultured on native collagen gels*

| ESAF Batch no. | "ESAF + filler" (μg/ml) Range tested | GSA | Maximum stimulation induced (% increase over the controls) |
|---|---|---|---|
| A-1* | 0·5–500 | 0·5–500 | 139 |
| A-2 | 2·5–500 | 25–50 | 89 |
| A-3* | 0·5–250 | 2·5 | 58 |
| A-4 | 2·5, 5 | 2·5, 5 | 31 |
| A-5* | 0·5–50 | 0·5–25 | 166 |
| A-6* | 0·05–50 | 2·5–25 | 77 |
| P-1 | 0·5–200 | 100 | 31 |
| P-2 | 0·05–5 | 0·5 | 61 |
| P-3 | 0·05–15 | 1 | 100 |

A low-mol.-wt angiogenic factor (ESAF) was isolated from rat Walker 256 carcinoma extracts by affinity chromatography ("A–" batches) and further gel filtration ("P–" batches) (Weiss, *et al.*, 1979). The actual concentration of ESAF is not known since an inert filler (albumin or lactose) was added to facilitate handling (see Methods). GSA was estimated by the increase in cell numbers in cultures of CBEC incubated with ESAF for 48–72 h. All ESAF batches were tested on cultures in plastic and native collagen; batches marked * were also tested on denatured collagen. GSA was apparent on native collagen only.

ESAF on cell growth are shown in Fig. 2. In this experiment, cells were placed on the different substrates and the medium changed after 24 h. The number of cells attached to the different substrates was then determined and all cultures received either 100 μl of BSS alone or BSS containing different amounts of ESAF. The cultures were then incubated at 37°C and cell numbers determined after 48 h. As can be seen in Fig. 2, no concentration of ESAF produced an increase in cell number on plastic dishes; the same results were also obtained with cells growing on gelatin films (not shown). However, when the cells were cultured on collagen gels, ESAF stimulated cell growth. A bell-shaped dose–response curve was obtained; in this case maximum stimulation was produced with 5 μg ESAF + filler/dish.

Results similar to those presented in Fig. 2 have been obtained with all the batches of ESAF tested and are summarized in the Table. The number of cells in the different cultures was determined 48–72 h after the addition of ESAF. The concentrations of ESAF producing growth stimulation usually fell within a narrow range. No effect of ESAF on cell proliferation on plastic or gelatin was ever observed. It should be noted that the actual concentration of ESAF used in these experiments was not known, as there was insufficient for weight determination and inert "fillers" (albumin or lactose) were used.

ESAF stimulated CBEC proliferation in growth medium containing either 8% or 16% FCS when the cells were cultured on native collagen, but not on plastic or denatured collagen. In the experiment shown in Fig. 3, the stimulation induced by 5 μg ESAF + filler/dish was more marked in 16% than in 8% FCS ($P < 0 \cdot 01$). The other concentrations of ESAF produced a similar increase in cell numbers irrespective of the serum concentration. Control experiments indicated that second passage CBEC which had been grown to confluence on native collagen (Schor *et al.*, 1979) behaved as first-passage cells in growth characteristics and response to ESAF, and

FIG. 3.—Growth-stimulatory activity of ESAF on CBEC in 8% or 16% serum concentration.

CBEC ($1 \cdot 1 \times 10^5$ cells/dish) were plated on native collagen in 8% or 16% FCS-medium. 24 h later the mediumwaschanged, the cells counted (shaded blocks) and ESAF (Batch A-5) added in the concentration shown. The cells in the different cultures were counted again 48 h after addition of ESAF (open blocks). 1, 5 and 50 μg "ESAF + filler"/dish stimulated cell growth ($P < 0{\cdot}01$); 100 μg/dish did not. Stimulation by 5 μg was greater with 16% than with 8% FCS ($P < 0{\cdot}01$). Cells plated on denatured collagen or on plastic and treated in the same way were not stimulated to proliferate (not shown).

FIG. 4.—Growth-stimulatory activity of ESAF on CBEC growing at different rates on native collagen.

CBEC in 8% FCS-medium were plated on collagen gels pre-incubated with serum-free medium (A: $9{\cdot}8 \times 10^4$ cells/dish), on gels pre-incubated with 16% FCS-medium (B: $9{\cdot}8 \times 10^4$ cells/dish) or on plastic dishes (C: $5 \times 10^4$ cells/dish). 24 h later the number of cells attached was determined (shaded blocks); the medium was changed and 5 μg of "ESAF + filler" (Batch A-3) in BSS (or BSS alone) were added. The cultures were returned to the incubator and the cells counted 48 h later (open blocks).

that the filler alone (albumin or lactose) had no effect on cell proliferation.

*Role of the substrate in the CBEC response to ESAF*

The mechanism by which ESAF stimulates CBEC proliferation in collagen gels is not understood. The following experiments were performed in order to determine whether cell proliferation is affected by the interaction of either ESAF or serum factors with the substrate.

The effects of preincubating the gels with serum are shown in Fig. 4. Collagen gels were incubated with either serum-free medium or medium containing 16% FCS for 24 h and then extensively washed by 6 changes of 2 ml BSS over a period of 24 h. CBEC in medium containing 8% serum was plated on these collagen gels and on plastic dishes. The medium on all cultures was changed 24 h later and replaced with fresh medium containing 8% FCS. Cell number was determined and all cultures then received 100 μl of BSS or BSS containing 5 μg of ESAF + filler. Cultures were incubated at 37°C and cell numbers determined after 48 h. As can be seen in Fig. 4, cell proliferation was greater on the gels pre-incubated with medium containing 16% FCS than on gels treated with serum-free medium. ESAF stimulated CBEC proliferation when the cells were growing on native collagen gels, irrespective of whether the growth rate was low (serum-free pre-incubated gels) or high (16% serum pre-incubated gels). Cultures

FIG. 5.—Growth-stimulatory activity of ESAF pre-incubated on different substrates.

Before seeding the cells, 1/6 of the dishes to be used (plastic, native and denatured collagen substrates) were incubated with 2 ml of serum-free medium containing 10 μg "ESAF + filler" (Batch A–6) per dish. The remaining dishes were incubated with 2 ml of serum-free medium. After 24 h at 37°C all dishes were washed × 8 with 2 ml BSS over a 20 h period. CBEC suspended in 16% FCS medium were then plated on to native collagen gels ($10^5$ cells/dish). 16 h later the number of cells attached was ($\times 10^4$) $3{\cdot}50 \pm 0{\cdot}18 \times 10^4$ and $3{\cdot}53 \pm 0{\cdot}17 \times 10^4$ on gels that had been pre-incubated with or without ESAF respectively (Day 0 in the graph). The medium with non-attached cells was then removed, 2 ml of fresh 16% FCS-medium were added per dish and the different cultures received 100 μl BSS or 10 μg "ESAF + filler" (Batch A–6) in 100 μl BSS. The solutions of ESAF had either been kept at −20°C or had been pre-exposed to plastic, native and denatured collagen by incubating 400 μg "ESAF + filler" per dish on the different substrates for 24 h before use (see text).

Controls (●) and gels pre-incubated with ESAF (○) received 100 μl BSS. The remaining cultures received an ESAF solution which had either been kept at −20°C (■) or had been pre-exposed to native collagen (△) to plastic (▲) or to denatured collagen (not shown). Cell growth on plastic and denatured collagen (not shown) was the same in all cultures. On native collagen the number of cells present in gels that had been pre-incubated with ESAF (○) was significantly higher than in the controls (●) on Days 1, 2 and 3 ($P < 0{\cdot}01$). Addition of the different ESAF solutions (△, ▲, ■) increased the cell numbers on Days 2 and 3 ($P < 0{\cdot}01$).

growing on plastic or gelatin films (not shown) were not stimulated by ESAF under the same conditions.

The results obtained in a similar experiment in which gels were preincubated with ESAF rather than serum are shown in Fig. 5. In this case, solutions of "pre-exposed" ESAF were prepared by incubating dishes containing the different substrates with a high concentration of ESAF in BSS for 24 h; this ESAF was then collected, diluted to the appropriate concentration (assuming no ESAF had been lost during the incubation) and stored at −20°C until required. Substrates were then prepared by incubating plastic dishes, gelatin films and native collagen gels for 24 h at 37°C with either serum-free medium or serum-free medium containing 10 μg of a particular batch of ESAF + filler (a concentration previously shown to stimulate cell growth on collagen gels). All substrata were then washed × 8 with BSS over a 20h period. CBEC in 16% serum medium were plated in these substrates and incubated for a further 16 h. The medium was changed and the cells attached to samples of all the substrates counted (Day 0). At this point cultures on substrates preincubated with serum-free medium received either 100 μl of BSS alone (controls), 100 μl of BSS containing 10 μg ESAF + filler (the same concentration used to pre-incubate the gels) or BSS containing 10 μg of ESAF + filler previously exposed to either plastic dishes, gelatin films or native collagen gels. Cells growing on substrates preincubated with ESAF received 100 μl of BSS only, with no additional ESAF. All cultures were then incubated for 3 more days. Cells growing in plastic dishes or gelatin films were not stimulated to grow more than the controls by ESAF in any of the conditions tested (data not shown). On native collagen gels, addition of the different ESAF solutions or preincubation of the gels with ESAF produced a similar increase in cell numbers after 3 days of incubation. In this particular experiment no lag period was observed on gels preincubated with ESAF, though this may be because cells on these gels had been in contact with it for 16 h when ESAF was added to the remaining cultures. In other experiments we have found that preincubation of the gels with ESAF does not shorten the lag period. These data indicate that ESAF binds to collagen and can stimulate CBEC proliferation in this state. Binding was not prevented by preincubation of the gels with 16% FCS before incubation with ESAF, and ESAF remained on the gels after extensive washing with BSS (for up to 48 h) or with 0·5M PBS (6 × 2ml) for 5 h.

The effects of ESAF pre-exposed to different substrates on CBEC proliferation suggest that it is not inactivated by exposure to plastic or denatured collagen, and that the cells must actually be growing on a native collagen substrate for ESAF to stimulate cell growth. This conclusion is consistent with the results obtained in other experiments in which ESAF had no effect on CBEC growing on plastic dishes with a collagen gel floating in the medium above the cells (data not shown). The floating collagen gels were either preincubated with ESAF or ESAF was added to the cultures at the same time as the gel.

*Effect of ESAF on fibroblasts*

Human embryo and adult skin fibroblasts showed no response to ESAF when tested under the same conditions as CBEC (data not shown).

### DISCUSSION

We have previously reported (Schor *et al.*, 1979) that the effect of TAF-containing tumour extracts on endothelial cells *in vitro* follows one of two patterns:

1. If the tumour extract was not trypsinized during the extraction procedure it would stimulate endothelial-cell (and fibroblast) proliferation both on plastic and native collagen substrates.
2. If the extraction was trypsinized it would stimulate endothelial cell proliferation only when the cells were cultured on native collagen and in the presence of platelet-released factor. Fibroblasts were not stimulated.

In view of the results presented here one can speculate that human platelet factors may act enzymatically upon TAF-containing tumour extracts, perhaps releasing an LMW angiogenic factor (ESAF) from a carrier protein. Trypsin, on the other hand, may destroy growth factors other than ESAF without affecting the latter.

ESAF is angiogenic when assayed on the chick chorioallantoic membrane (Weiss *et al.*, 1979) and we have shown that it stimulates endothelial-cell (CBEC) proliferation on native collagen gels *in vitro* without addition of human platelet factors to the cultures. Unlike some TAF-containing tumour extracts (Schor *et al.*, 1979) ESAF does not stimulate fibroblast growth.

The data presented in Fig. 3 indicate that the response of the endothelial cells to ESAF can be influenced by the concentrations of serum in the medium. We are currently investigating whether this effect is due to bovine platelet factors in the serum. It has been shown, however, that growth factors derived from human serum and platelets are immunologically different from bovine serum factors (Antoniades & Scher, 1978).

The stimulation of CBEC proliferation on native collagen gels *in vitro* by all of the batches of ESAF tested (9 in total) shows a similar bell-shaped dose–response curve. CBEC cultured on either plastic culture dishes or denatured collagen films showed no response to ESAF under any of the conditions tested.

The concentration of ESAF which induces the maximal response is not yet known since the amounts obtained were so small that weighing was not feasible. The incorporation of inert "fillers" during the ESAF extraction procedure facilitated its handling. The amount of ESAF which stimulated endothelial-cell proliferation was between 1/100 to 1/1,000 the amount used to detect angiogenic activity in the chick choriollantoic membrane assay.

Although fewer cells remain attached to the collagen gels than to the plastic dishes 24 h after plating, it is unlikely that an ESAF-sensitive subpopulation of cells is selected, since stock cultures of CBEC grown on collagen gels show exactly the same growth characteristics and response to ESAF when subsequently cultured on plastic dishes and collagen gels as reported here.

ESAF does not affect cell attachment to the substratum.

Why a native collagen substrate is required for ESAF to stimulate CBEC proliferation is not known. Our results suggest that the slower growth rate on native collagen than on denatured collagen or plastic is due to adsorption of serum growth factors to the gel. By manipulating the serum concentration available to the cells or pre-incubating the gels with serum-containing medium, CBEC could be grown at similar rates on plastic and native collagen, and our results show that stimulation of cell growth by ESAF occurs only on native collagen substrates, independently on the growth rate of the control cultures.

Unlike stimultaion by increasing serum concentrations, ESAF-stimulated and unstimulated cultures reached the same cell saturation density. Lack of stimulation by ESAF of cultures on plastic and gelatin does not appear to be due to inactivation of ESAF on those substrates, since ESAF pre-exposed to plastic or gelatin was still active.

ESAF binds to native collagen, and this may be a crucial step on its mode of action. Our data show that ESAF-bound collagen stimulates cell proliferation only when used as the substrate for the cells. ESAF bound to collagen was not removed by extensive washing with 0·5M PBS, thus the binding does not seem to be a simple electrostatic attachment.

The importance of the extracellular matrix in cell behaviour is widely recognized. Collagen is a major constituent of the extracellular matrix and it has been shown to affect cell attachment (Schor & Court, 1979; Klebe, 1975; Murray *et al.*, 1979) proliferation (Liotta *et al.*, 1978; Schor, 1980; Rath & Reddi, 1979; Gey *et al.*, 1974; Ehrman & Gey, 1956) migration (Algire & Chalkley, 1945; Kadish *et al.*, 1979), differentiation (Reddi & Anderson, 1976; Meier & Hay, 1975; Konigsberg & Hauschka, 1965) morphology (Gospodarowicz *et al.*, 1978) and collagen biosynthesis (Meier & Hay, 1974). New formation of collagen, as well as neovascularization, are required for continuous tumour growth; both the tumour-associated collagen and blood vessels are produced by the host (Folkman 1974; Gullino, 1973). It is therefore possible that a native collagen substrate allows the CBEC to react to ESAF as they would *in vivo*, whereas more artificial substrates such as plastic or denatured collagen do not. Chemical identification of the many angiogenic factors reported in the literature (Folkman *et al.* 1971;, Phillips *et al.*, 1976; McAuslan & Hoffman, 1979; Polverini *et al.*, 1977; Wolf & Harrison, 1973; Klagsburn *et al.*, 1976; Auerbach *et al.*, 1976; Huseby *et al.*, 1975; Maiorana & Gullino, 1978; Tsukamoto & Sugino, 1979; Gospodarowicz & Thakral, 1978) including our own will determine whether they are the same. Published data suggest that TAF is a unique tumour marker (Algire & Chalkley, 1945; Folkman, 1974; Phillips *et al.*, 1976; Maiorana & Gullino, 1978) but it may be the same as other angiogenic factors, possibly common to all tissue capable of growth and repair, its concentration related to the metabolic activity of the tissue. It is worth pointing out that the angiogenic factor used in our experiments (ESAF) was in fact tumour-derived. It will be interesting to know whether the reported lack of stimulatory activity by other purified angiogenic factors when tested on endothelial cells *in vitro* (McAuslan & Hoffman, 1979; Folkman, in Kumar, 1980) does also apply when the cells are cultured on native collagen, and whether endothelial cells derived from capillaries and from large vessels can be stimulated in a similar way by ESAF in culture.

This work was supported by the Cancer Research Campaign.

### REFERENCES

ANTONIADES, H. N. & SCHER, C. D. (1978) Growth factors derived from human serum, platelets and pituitary: Properties and immunologic cross-reactivity. *Natl Cancer Inst., Monogr.*, **48**, 137.

ALGIRE, G. H. & CHALKLEY, H. W. (1945) Vascular reactions of normal and malignant tissue *in vivo*. 1. Vascular reactions of mice to wounds and to normal and neoplastic transplants. *J. Natl Cancer Inst.*, **6**, 73.

AUERBACH, R., KUBAI, L. & SIDKY, Y. (1976) Angiogenesis induction by tumours, embryonic tissues, and lymphocytes. *Cancer Res.*, **36**, 3435.

AUSPRUNK, D. H. & FOLKMAN, J. (1977) Migration and proliferation of endothelial cells in preformed and newly formed vessels during tumour angiogenesis. *Microvasc. Res.*, **14**, 53.

BROWN, R. A., WEISS, J. B., TOMLINSON, I. W., PHILLIPS, P. & KUMAR, S. (1980) An angiogenic factor from synovial fluids resembling that from tumours. *Lancet*, i, 682.

CAVALLO, T., SADE, R., FOLKMAN, J. & COTRAN, R. S. (1973) Endothelial regeneration: Ultrastructural and autoradiographic studies of the early proliferative response in tumour angiogenesis. *Am. J. Pathol.*, **70**, 345.

EHRMANN, R. L. & GEY, G. O. (1956) The growth of cells on a transparent gel of reconstituted rat-tail collagen. *J. Natl Cancer Inst.*, **16**, 1375.

FENSELAU, A. & MELLO, R. J. (1976) Growth stimulation of cultured endothelial cells by tumour cell homogenates. *Cancer Res.*, **36**, 3269.

FOLKMAN, J. (1974) Tumour angiogenesis, *Adv. Cancer Res.*, **19**, 331.

FOLKMAN, J. & COTRAN, R. S. (1976) Relation of vascular proliferation to tumour growth. *Int. Rev. Exp. Pathol.*, **16**, 207.

FOLKMAN, J., MERLER, E., ABERNATHY, C. & WILLIAMS, G. (1971) Isolation of a tumour factor responsible for angiogenesis. *J. Exp. Med.*, **133**, 275.

GEY, G. O., SVOTELIS, M., FOARD, M. & BANG, F. B. (1974) Long term growth of chicken fibroblasts on a collagen substrate. *Exp. Cell Res.*, **84**, 63.

GIMBRONE, M. A., COTRAN, R. S. & FOLKMAN, J. (1974) Tumour growth and neovascularization: An experimental model using the rabbit cornea. *J. Natl Cancer Inst.*, **53**, 413.

GIMBRONE, M. A., JR & GULLINO, P. M. (1976) Neovascularization induced by intraocular xenografts of normal, preneoplastic and neoplastic mouse mammary tissues. *J. Natl Cancer Inst.*, **56**, 305.

GOSPODAROWICZ, D., GREENBURG, G. & BIRDWELL, C. R. (1978) Determination of cellular shape by the extracellular matrix and its correlation with the control of cellular growth. *Cancer Res.*, **38**, 4155.

GOSPODAROWICZ, D. & THAKRAL, K. (1978) Production of a corpus luteum angiogenic factor responsible for proliferation of capillaries and neovascularization of the corpus luteum. *Proc. Natl Acad. Sci.*, **75**, 847.

GULLINO, P. M. (1973) Regression of solid tumours. In *Chemotherapy of Cancer Dissemination and Metastasis*. Eds Garattini & G. Franchi. New York: Raven Press. p. 89.

HUSEBY, R. A., CURRIE, C., LAGERBORG, V. A. & GARB, S. (1975). Angiogenesis about and within grafts of normal testicular tissue: A comparison with transplanted neoplastic tissue. *Microvasc. Res.*, **10**, 396.

KADISH, J. L., BUTTERFIELD, C. E. & FOLKMAN, J. (1979) The effect of fibrin on cultured endothelial cells. *Tissue Cell*, **11**, 99.

KLAGSBURN, M., KNIGHTON, D. & FOLKMAN, J. (1976) Tumour angiogenesis activity in cells grown in tissue culture. *Cancer Res.*, **36**, 110.

KLEBE, R. J. (1975) Cell attachment to collagen: the requirement for energy. *J. Cell Physiol.*, **86**, 231.

KONIGSBERG, I. R. & HAUSCHKA, S. D. (1965) Cell and tissue interaction in the reproduction of cell type. In *Reproduction: Molecular, Subcellular and Cellular*. Ed. Locke. N.Y.: Academic Press. p. 243.

KUMAR, S. (1980) Angiogenesis and anti-angiogenesis. *J. Natl. Cancer Inst.*, **64**, 683.

LIOTTA, L. A., VEMBU, D., KLEINMAN, H. K., MARTIN, G. R. & BOONE, C. (1978) Collagen required for proliferation of cultured connective tissue cells, but not their transformed counterparts. *Nature*, **272**, 622.

MAIORANA, A. & GULLINO, P. M. (1978) Acquisition of angiogenic capacity and neoplastic transformation in the rat mammary gland. *Cancer Res.*, **38**, 4409.

MCAUSLAN, B. R. & HOFFMAN, H. (1979) Endothelium stimulating factor from Walker carcinoma cells. Relation to tumour angiogenic factor. *Exp. Cell Res.*, **119**, 181.

MEIER, S. & HAY, E. D. (1974) Control of corneal differentiation by extracellular materials. Collagen as a promotor and stabilizer of epithelial stroma production. *Dev. Biol.*, **38**, 249.

MEIER, R. S. & HAY, E. D. (1975) Stimulation of corneal differentiation by interaction between cell surface and extracellular matrix. *J. Cell Biol.*, **66**, 275.

MURRAY, J. C., STINGL, G., KLEINMAN, H. K., MARTIN, G. R. & KATZ, S. I. (1979) Epidermal cells adhere preferentially to type IV (basement membrane) collagen. *J. Cell Biol.*, **80**, 197.

PHILLIPS, P., KUMAR, P., KUMAR, S. & WAGHE, M. (1979) Isolation and characterization of endothelial cells from rat and cow brain white matter. *J. Anat.*, **129**, 261.

PHILLIPS, P. & KUMAR, S. (1979) Tumour angiogenesis factor (TAF) and its neutralization by an xenogeneic antiserum. *Int. J. Cancer*, **23**, 82.

PHILLIPS, P. J., STEWARD, J. K. & KUMAR, S. (1976) Tumour angiogenesis factor (TAF) in human and animal tumours. *Int. J. Cancer*, **17**, 549.

POLVERINI, P. J., COTRAN, R. S., GIMBRONE, M. A., JR & UNANUE, E. R. (1977) Activated macrophages induce vascular proliferation. *Nature*, **269**, 804.

RATH, N. C. & REDDI, A. H. (1979) Collagenous bone matrix is a local mitogen. *Nature*, **278**, 855.

REDDI, A. H. & ANDERSON, W. A. (1976) Collagenous bone matrix-induced endochondrial ossification and haemopoiesis. *J. Cell Biol.*, **69**, 557.

SCHOR, S. L. (1980) Cell proliferation and migration on collagen substrata *in vitro*. *J. Cell Sci.*, **41**, 159.

SCHOR, S. L. & COURT, J. (1979) Different mechanisms in the attachment of cells to native and denatured collagen. *J. Cell Sci.*, **38**, 267.

SCHOR, A. M., SCHOR, S. L. & KUMAR, S. (1979) Importance of a collagen substratum for stimulation of capillary endothelial cell proliferation by tumour angiogenesis factor. *Int. J. Cancer*, **24**, 225.

TSUKAMOTO, K. & SUGINO, Y. (1979) Tumour angiogenesis activity in clonal cells transformed by bovine adenovirus Type 3. *Cancer Res.*, **39**, 1305.

WEISS, J. B., BROWN, R. A., KUMAR, S. & PHILLIPS, P. (1979) An angiogenic factor isolated from tumours: a potent low molecular weight compound. *Brit. J. Cancer*, **40**, 493.

WOLF, J. E., JR & HARRISON, R. G. (1973) Demonstration and characterization of an epidermal angiogenic factor. *J. Invest. Dermatol.*, **61**, 130.